# Polyaniline‐Coated Mesoporous Carbon Nanosheets with Fast Capacitive Energy Storage in Symmetric Supercapacitors

**DOI:** 10.1002/advs.202301923

**Published:** 2023-05-10

**Authors:** Jungchul Noh, Suk Jekal, Chang‐Min Yoon

**Affiliations:** ^1^ McKetta Department of Chemical Engineering and Texas Material Institute The University of Texas at Austin Austin TX 78712 USA; ^2^ Department of Chemical and Biological Engineering Hanbat National University 125 Dongseo‐daero, Yuseong‐gu Daejon 34158 South Korea

**Keywords:** mesoporous carbon, nanocrystal, polyaniline, pseudocapacitance, symmetric supercapacitors

## Abstract

Polyaniline‐capped mesoporous carbon nanosheets with high conductivity and porosity are synthesized by vapor deposition polymerization. The mesoporous carbon template is prepared by removing ordered cubic iron oxide nanocrystals embedded in the carbon matrix obtained by thermal decomposition of an iron‐oleate complex in a sodium chloride matrix. The evaporated aniline monomers are slowly polymerized on the carbon surface pretreated with FeCl_3_ as an initiator, partially filling the carbon pores to improve conductivity. The resulting products exhibit efficient hybrid energy storage mechanisms of electric double‐layer capacitance and pseudocapacitance. When the nanosheets are assembled for a symmetric supercapacitor, the device capacitance reaches 107.8 F g^−1^, at a current density of 0.5 A g^−1^, and a capacitance retention of 69.6% is achieved at a ten times higher current density of 5 A g^−1^. Electrochemical impedance spectroscopy reveals that the transition from resistive to capacitive behavior occurs within 0.63 s, indicating that fast ion and charge transport results in high capacitance and rate capability. The corresponding energy and power densities are 9.59 Wh kg^−1^ and 200.1 W kg^−1^ at a current density of 0.5 A g^−1^, demonstrating efficient energy storage in a symmetric supercapacitor.

## Graphical abstract

The highly ordered mesoporous carbon (mC) matrix is synthesized and polyaniline (PANI) is partially filled with the vapor deposition polymerization method. The resulting mC/PANI nanosheets‐assembled symmetric supercapacitor exhibits energy density and power density of 9.59 Wh kg^−1^ and 200.1 W kg^−1^ at a current density of 0.5 A g^−1^, which are higher than the majority of the reported symmetric supercapacitors.

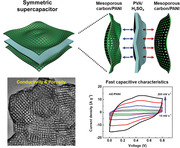

## 1 Introduction

Supercapacitors have been widely investigated for use as power supplies to complement batteries owing to their fast charge–discharge capability and long cycle life.^[^ ^1^, ^2^ ^]^ Their operation mainly relies on carbon materials that exhibit electric double‐layer capacitance (EDLC); hybridization with redox reactions is possible, but EDLC‐type materials are required for fast charge supply.^[^ ^3^, ^4^, ^5^ ^]^ Under an applied voltage, charges are separated with opposing polarity forms, one at the electrode surface and the other in the electrolytes. Thus, increasing the surface area and conductivity improves the efficiency of the energy storage mechanism.^[^ ^6^, ^7^ ^]^ With an increase in the number of active sites where electrolyte adsorption occurs, more charges are accumulated.^[^ ^8^ ^]^ Conductive surfaces boost electrolyte transport for fast energy delivery.^[^ ^9^ ^]^ Various carbon materials including graphene, carbon nanofibers, and porous carbon have been extensively studied with an aim to realize high energy and power densities.^[^ ^10^, ^11^, ^12^, ^13^ ^]^

Mesoporous carbon (mC) nanosheets are promising energy‐storage materials owing to their high surface area, tunable pore size, adjustable pore symmetry, and well‐defined porous channels.^[^ ^14^, ^15^ ^]^ These pores provide charge accumulation sites for high energy storage capability and enable electrolytes to be easily accessible to surface atoms with a short diffusion distance.^[^ ^16^ ^]^ The restacking of nanosheets often blocks active sites; however, this issue has been significantly mitigated.^[^ ^17^ ^]^ Furthermore, the porous structure reduces the electrical conductivity because of the broken symmetry of sp^2^ hybridized carbon, resulting in the loss of active sites and slow electrolyte transport.^[^ ^18^, ^19^ ^]^ This trade‐off has motivated researchers to determine an optimal porosity using various templates such as mesoporous silica,^[^ ^20^ ^]^ silica inverse opal,^[^ ^21^ ^]^ anodic aluminum oxide,^[^ ^22^ ^]^ and block copolymer micelles.^[^ ^23^ ^]^

Nanocrystal assemblies interconnected by organic molecules can be used as mesoporous carbon templates.^[^ ^24^, ^25^ ^]^ After calcination and etching of nanocrystal cores, organic ligands turn into graphitic carbons at relatively low calcination temperatures (<1500 °C) owing to the presence of metallic or metal oxide cores as graphitization catalysts.^[^ ^26^, ^27^, ^28^ ^]^ However, the resulting products are partially graphitic or semigraphitic and cannot utilize the entire surface for energy storage.^[^ ^29^, ^30^, ^31^ ^]^ Complete graphitization can be achieved by heating the mesoporous carbon at higher temperatures (>2000 °C), which unfortunately leads to structural collapse with the loss of surface area and ordered pores.^[^ ^32^, ^33^ ^]^ The synthetic difficulty to produce conductive and porous carbons using the bottom‐up approach has limited their use in supercapacitor applications. Thus, the ability to make well‐defined porous channels and post‐treatment for high conductivity will open opportunities in the energy storage system.

The introduction of conducting polymers is an alternative approach. In particular, polyaniline (PANI) has drawn significant attention owing to its high conductivity, fast Faradaic reaction, and high theoretical capacitance (2000 F g^−1^).^[^ ^34^, ^35^ ^]^ PANI can form an expanded coil conformation on carbon materials for compact packing between polymer chains, thereby increasing electrical conductivity.^[^ ^36^, ^37^, ^38^ ^]^ In addition, the redox reactions of PANI offer an additional pseudocapacitive contribution. The Faradaic transition among the three forms (leucoemeraldine, emeraldine, and pernigraniline) increases the energy storage capability.^[^ ^39^, ^40^ ^]^ However, unlike the stable EDLC mechanism, repeated redox reactions often cause structural damage, with poor cyclability and rate capability.^[^ ^41^ ^]^ A porous electrochemical surface with facile movement of electrolytes can minimize the damage and achieve a stable electrochemical response.^[^ ^42^ ^]^ These are highly desired for the current trends of sustainable and integrable device architectures, including flexible and self‐healable devices, in‐plane supercapacitors, and integration of energy conversion and energy storage systems.^[^ ^43^, ^44^, ^45^, ^46^, ^47^, ^48^ ^]^ Although the structural benefits are expected, a controlled PANI coating with the retention of porous structures has not been achieved because fast polymerization in a liquid phase normally produces composite mixtures. As the pore size decreases, it becomes more challenging to partially clog the pores of carbons with conducting polymers. Therefore, the synthetic success will provide insight into the incorporation of conducting polymers into a variety of mesoporous carbons for future supercapacitors.

Herein, we report the synthesis of PANI‐coated mC nanosheets for conductive and porous frameworks that exhibit high energy storage capability. Heating an iron‐oleate complex in a sodium chloride matrix produces cubic iron oxide (Fe_2_O_3_) nanocrystals interconnected by carbon networks (Fe_2_O_3_/C). The removal of nanocrystals by acid treatment left the mC nanosheets. Subsequently, PANI was coated onto the nanosheets by vapor deposition polymerization (VDP) to achieve high porosity and conductivity. The resulting PANI‐coated mC (mC/PANI) nanosheets exhibited a high capacitance of 469.2 F g^−1^ at a current density of 0.5 A g^−1^ in a three‐electrode system. When the electrodes are assembled as a symmetric supercapacitor, the device capacitance reaches 107.8 F g^−1^ at a current density of 0.5 A g^−1^ with 69.6% capacitance retention at 5 A g^−1^. The energy density and power density were found to be 9.59 Wh kg^−1^ and 200.1 W kg^−1^, respectively, at a current density of 0.5 A g^−1^. Using electrochemical impedance spectroscopy, the fast transition from resistive to capacitive behavior was studied to confirm the efficient ion and charge transport properties of the device.

## 2 Results and Discussion

### 2.1 Fabrication of Mesoporous Carbon Nanosheets and Incorporation of Polyaniline

**Figure 1:**
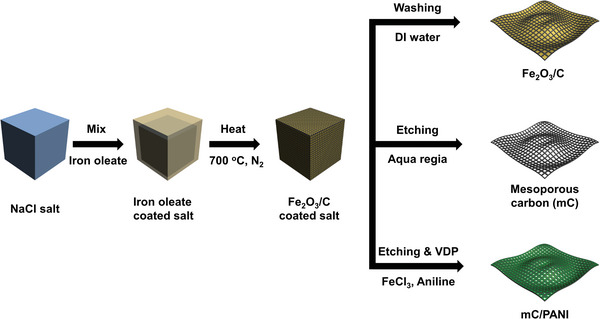
Schematic illustration of the synthesis of Fe_2_O_3_/C, mC, and mC/PANI nanosheets. Fe_2_O_3_/C‐coated NaCl salt was prepared by heating iron oleate in NaCl at 700 °C under nitrogen atmosphere. The following dissolution of NaCl with water produced Fe_2_O_3_/C nanosheets. The removal the nanocrystal cores with aqua regia resulted in mC nanosheets. PANI coating on the mC nanosheets was performed using the vapor deposition polymerization method.

**Figure** **1** illustrates the synthetic procedure to prepare mC/PANI nanosheets. For the template, Fe_2_O_3_/C nanosheets are prepared by heating an iron‐oleate complex in a sodium chloride (NaCl) matrix under a nitrogen atmosphere. Iron oleate thermally decomposes to form iron oxide nanocrystals and the remaining oleate forms carbon frameworks that interconnect nanocrystals on the NaCl powder surface. Washing the products with water dissolves the NaCl, forming 2D carbon nanosheets embedded with iron oxide nanocrystals. A strong acid treatment with aqua regia removes nanocrystal cores and produces mC nanosheets. In the final step, PANI is incorporated into the mC nanosheets by vapor deposition polymerization. To retain the porous structure, a controlled monomer supply is required such that polymeric nucleation occurs only on the carbon surface without secondary nucleation. In general, the activation energy for heterogeneous nucleation is lower than that for homogeneous nucleation.^[^ ^49^ ^]^ Thus, mild reaction conditions are favorable for the successful incorporation of incoming materials on the target surface.^[^ ^50^, ^51^ ^]^ Aniline monomers evaporated under vacuum at 60 °C are slowly polymerized on the surface of the mC nanosheets presoaked with ferric chloride (FeCl_3_) as an initiator. The resulting PANI partially fills the holes, but does not block the entire porosity.

**Figure 2:**
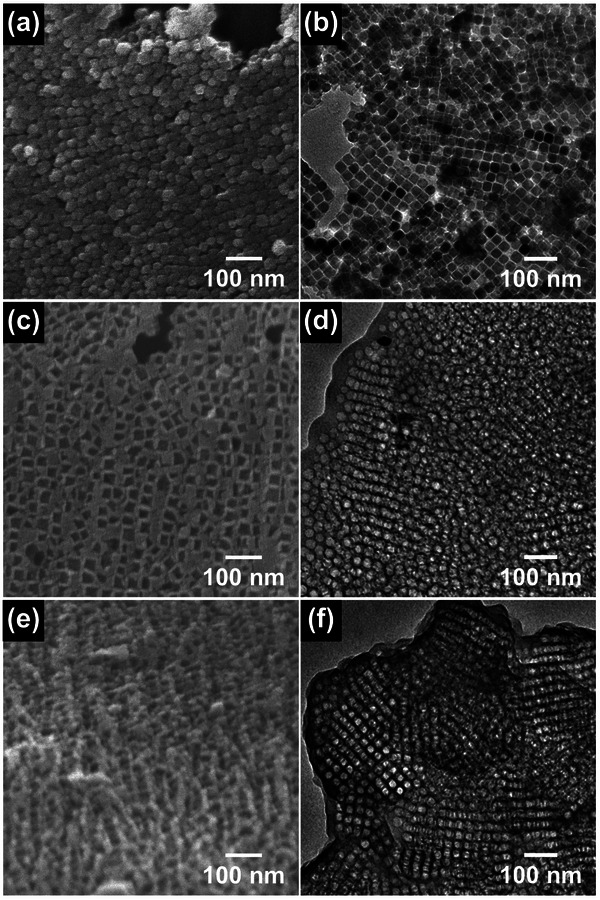
SEM and TEM images of a,b) Fe_2_O_3_/C, c,d) mC, and e,f) mC/PANI nanosheets. The Fe_2_O_3_ nanocrystals embedded in carbon have a cubic shape with an average edge length of 40 ± 3 nm. The removal of nanocrystals using aqua regia produces mC nanosheets. PANI coating on the mC nanosheets results in a rough surface with partially filling the pores.

**Figure** **2** shows scanning electron microscopy (SEM) and transmission electron microscopy (TEM) images of the Fe_2_O_3_/C, mC and mC/PANI nanosheets. The iron oxide nanocrystals obtained from the decomposition of iron oleate in the NaCl matrix are nearly perfect cubes with an average edge length of 40 ± 3 nm. The addition of NaCl is important for forming a cubic shape with a tight size distribution. Na‐oleate molecules can be generated from the Fe‐(oleate)_3_ complex by a counterion exchange reaction during heating.^[^ ^52^, ^53^ ^]^ Subsequently, Na‐oleate molecules modify the surface energy by exposing the polar facets of the nanocrystals,^[^ ^54^ ^]^ resulting in a cubic nanocrystal shape. The thermal decomposition of iron oleate without NaCl produces nanocrystals with an irregular shape and extremely broad size distribution of 10–200 nm (Figure S1, Supporting Information).

Together with the addition of NaCl, the heating rate used for growth also determines the ordering of nanocrystals by changing the conversion yields of carbon (Figure S2, Supporting Information). When the iron‐oleate complex is decomposed at lower heating rates, more carbon is obtained with a large distance between the particles. Conversely, a significant loss of carbon at a higher heating rate leads to the agglomeration of nanocrystals. The ordered assembly of nanocrystals in carbon matrix is produced at an optimal heating rate of 10 °C min^−1^, although the ordering domain area is on the scale of a few square micrometers. When the assembled nanocrystals are etched out, mC nanosheets are produced and complete removal can be achieved by a strong acid treatment. The carbon originally capping nanocrystals is not destroyed by the etchant, resulting in ordered porosity. VDP of aniline onto the mC nanosheets generates a highly rough surface. The Brunauer–Emmett–Teller (BET) surface area estimated from the N_2_ isotherms changes from 161 to 46 m^2^ g^−1^, indicating partial pore clogging by PANI (Figure S3, Supporting Information). The contrast change in the TEM image is also in good agreement with the successful incorporation of PANI into the mC template.

**Figure 3:**
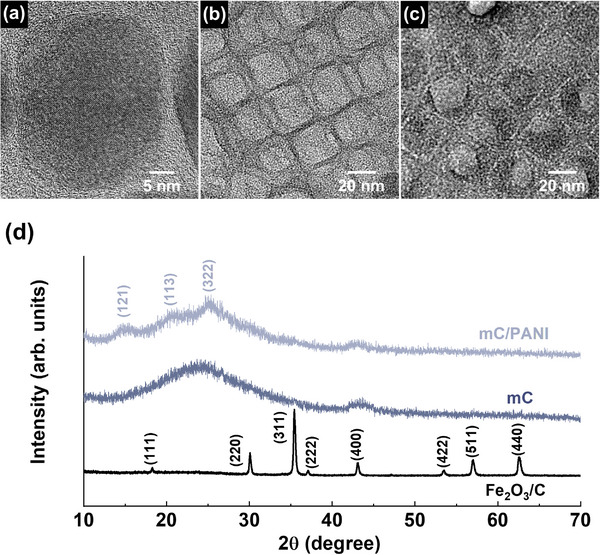
HR‐TEM images of a) Fe_2_O_3_/C, b) mC, and c) mC/PANI nanosheets. The *d*‐spacing of 0.29 nm in (a) corresponds to the (220) crystal plane of Fe_2_O_3_ nanocrystals. No crystalline structure is observed after the complete removal of the nanocrystals. PANI coating on the mC nanosheets partially fills the porous structure. d) XRD of Fe_2_O_3_/C, mC, and mC/PANI nanosheets. The diffraction pattern of iron oxide nanocrystals corresponds to maghemite *γ*‐Fe_2_O_3_.

High‐resolution transmission electron microscopy (HR‐TEM) and X‐ray diffraction (XRD) revealed the morphology and crystal structure of the Fe_2_O_3_/C, mC, and mC/PANI nanosheets (**Figure** **3**). In cubic iron oxide nanocrystals, the *d*‐spacing (lattice spacing) of 0.29 nm measured by HR‐TEM is in agreement with the distance between (220) planes in Fe_2_O_3_ (Figure S4, Supporting Information). Moreover, the XRD pattern of the iron oxide matches that of maghemite *γ*‐Fe_2_O_3_ (JCPDS # 98‐008‐7119).^[^ ^55^ ^]^ The average (111), (220), and (311) *d*‐spacings were 0.49, 0.30, and 0.25 nm, respectively, corresponding to a lattice constant of 0.84 nm, which is close to the value reported for maghemite *γ*‐Fe_2_O_3_ (0.835 nm).^[^ ^56^ ^]^ The crystallinity of iron oxide completely disappears by the etching of the nanocrystal cores and the remaining pores follow a cubic nanocrystal shape with an edge length of 40 nm. The pores were then partially filled with PANI using VDP. The crystallographic lattice of mC/PANI was not observed using HR‐TEM or the corresponding selected‐area electron diffraction ( Figure S5, Supporting Information). However, the XRD pattern of the mC/PANI nanosheets exhibited three peaks at 2*θ* = 15.3, 20.6, and 25.4 (*d*‐spacings = 0.58, 0.43, and 0.35 nm), revealing the semicrystalline nature of PANI. In particular, the peak at 25.4 is the face‐to‐face *π* −*π* stacking distance.^[^ ^57^ ^]^ The peak at 25.4 on the carbon background suggested the *π* −*π* stacking between the basal plane of graphitic carbon in mC and the quinoid rings of PANI. This interaction leads to an expanded coil conformation of PANI chains on the carbon surface. The compact packing of PANI inhibits significant ring rotations (*π*‐conjugation defects) of the phenyl rings, resulting in an increase in charge delocalization along the backbone chains.^[^ ^38^ ^]^

**Figure 4:**
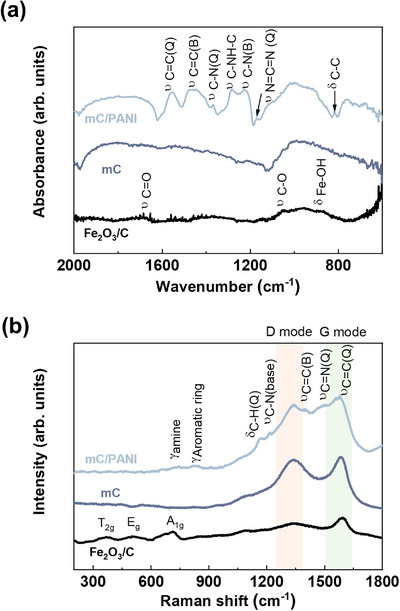
a) FTIR and b) Raman spectra of Fe_2_O_3_/C, mC, and mC/PANI nanosheets. The complementary vibrations in the spectra show the successful VDP of PANI onto mC nanosheets obtained by etching the *γ*‐Fe_2_O_3_ embedded in partially graphitic carbon. Changes in the Raman signal due to laser exposure (532 nm, 10 mW) were avoided by acquiring the spectra immediately after the samples were exposed to the laser. In the parentheses, B and Q denote the benzenoid and quinoid forms of PANI. The shaded green and red colors represent G and D bands of carbon.

**Figure** **4**a shows the Fourier‐transform infrared (FTIR) spectra of the nanosheets. The spectrum of Fe_2_O_3_/C exhibits stretching vibrations of C=O, C—O, and Fe—OH at 1677, 1055, and 867 cm^−1^,^[^ ^58^ ^]^ respectively, indicating that the iron oxide nanocrystals are embedded in the partially graphitic carbon matrix. The spectrum becomes nearly featureless after the removal of the nanocrystals. However, the spectrum of the mC/PANI nanosheets showed multiple peaks, including the quinoid stretching vibrations of C=C (1555 cm^−1^), C—N (2634 cm^−1^), and N=C=N (2856 cm^−1^), benzenoid stretching vibrations of C=C (1455 cm^−1^) and C—N (1228 cm^−1^), and the stretching mode of C—NH—C in the base emeraldine form (1281 cm^−1^).^[^ ^59^, ^60^, ^61^ ^]^

The Raman spectra show complementary vibrational peaks for the nanosheets (Figure 4b). The spectrum of Fe_2_O_3_/C exhibits T_2g_, E_g_, and A_1g_ Raman active phonon modes at 365, 511, and 700 cm^−1^, respectively, confirming the crystal phase of maghemite *γ*‐Fe_2_O_3_.^[^ ^62^ ^]^ Raman peaks also appear near the expected G and D bands at 1580 and 1350 cm^−1^, resulting from the in‐plane vibrations of sp^2^‐hybridized carbon and out‐of‐plane vibrations from structural defects, respectively.^[^ ^63^ ^]^ Thus, the spectrum indicated the formation of maghemite *γ*‐Fe_2_O_3_ in partially graphitized carbon. The intensity ratio of the D/G bands increases with the etching of nanocrystals, which is consistent with the structural defects caused by porosity. The spectrum of mC/PANI shows additional PANI vibrations on the carbon background, including the quinoid stretching vibrations of C=C (1580 cm^−1^) and C=N (1485 cm^−1^), stretching vibrations of benzenoid rings (1401 cm^−1^), C—H bending vibration of quinoid rings (1164 cm^−1^), stretching vibration of the C—N bond in the emeraldine form (1215 cm^−1^), benzene ring deformation (820 cm^−1^), and amine deformation from the bipolaronic form of emeraldine salt (740 cm^−1^).^[^ ^59^, ^64^ ^]^ The FTIR and Raman spectra confirmed the successful VDP of PANI onto mC nanosheets obtained by etching the *γ*‐Fe_2_O_3_ embedded in partially graphitic carbon.

**Figure 5:**
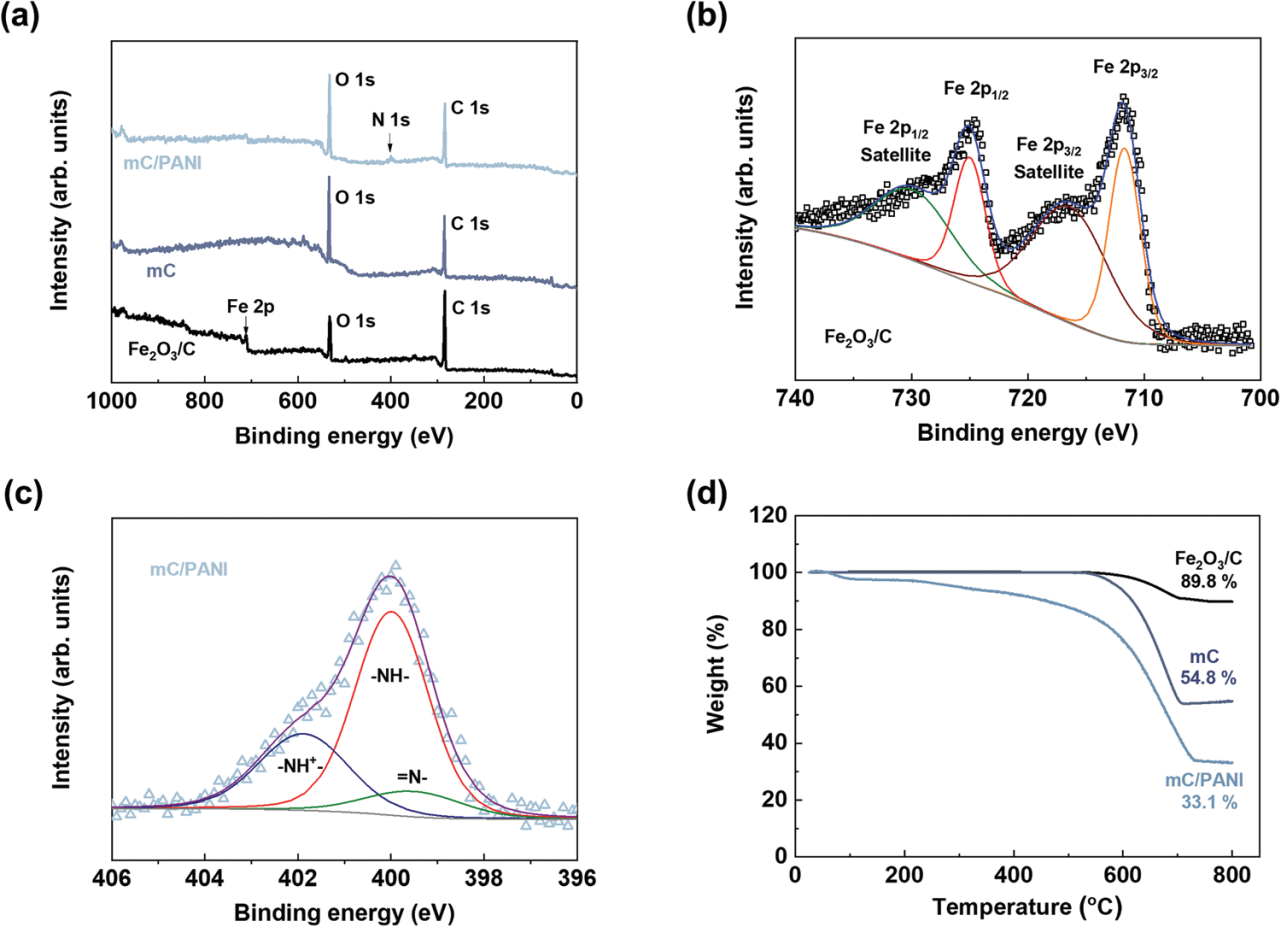
a) Survey scan of XPS for Fe_2_O_3_/C, mC, and mC/PANI nanosheets. b) Fe 2p XPS of Fe_2_O_3_/C sample. c) N 1s XPS of mC/PANI nanosheets. d) TGA of XPS for Fe_2_O_3_/C, mC, and mC/PANI nanosheets.

X‐ray photoelectron spectroscopy (XPS) revealed the surface characteristics of the nanosheets (**Figure** **5**a–c). The XPS spectra confirm that Fe in Fe_2_O_3_/C disappears after the removal of the core and N appears upon the addition of PANI. Fe 2p peaks exhibit 2p_3/2_ (711.7 eV) and 2p_1/2_ (725.1 eV) spin–orbit splitting of 13.4 eV, along with satellite peaks at 716.8 and 730.2 eV, which is consistent with *γ*‐Fe_2_O_3_ surface states.^[^ ^65^ ^]^ The N 1s peak spectrum was also deconvoluted into doped amine (401.8 eV), doped imine (400.0 eV) and undoped amine (399.4 eV), resulting from the PANI oxidation states.^[^ ^66^ ^]^ The peaks for C and O, commonly observed in every sample, indicate the presence of either residual oleate molecules or oxidation of carbon in the defect sites. The C/O ratio decreased owing to the etching process because the porous structure generated more defect sites that were sensitive to oxidation.

Thermogravimetric analysis (TGA) was performed to study the compositional ratio of the nanosheets (Figure 5d). In an inert N_2_ atmosphere, an increase in weight from oxidation was not observed from room temperature to 800 °C. After heating, the weight of the Fe_2_O_3_/C, mC, and mC/PANI nanosheets remained at 89.8, 54.8, and 33.1 wt%, respectively. The minor weight change of the Fe_2_O_3_/C sample occurs because the nanocrystals and graphitic carbon were relatively stable under heating conditions. The increase in the weight loss of the mC nanosheets was attributed to the pyrolysis of the oxygen‐containing groups (e.g., —OH, —COOH, etc.) in the defect sites, which is consistent with the decrease in the G/D bands in the Raman spectra and the C/O ratio in the XPS spectra. In addition, the amount of PANI on the mC template was determined from the remaining weight difference between mC and mC/PANI. The calculation confirms that the PANI content in the nanosheets is ≈31.7 wt%.

### 2.2 Electrochemical Properties in a Three‐Electrode System

The electrochemical properties of the Fe_2_O_3_/C, mC, and mC/PANI nanosheets were studied using a three‐electrode system to evaluate their applicability in supercapacitor devices. The three‐electrode configuration was composed of the active materials as the working electrode, Pt wire as the counter electrode and Ag/AgCl as the reference electrode in 1 m KOH or H_2_SO_4_ electrolyte solution. A base electrolyte was used for Fe_2_O_3_/C to avoid the degradation of metal oxide nanocrystals and an acid solution was applied to the mC and mC/PANI nanosheets. The working electrode was prepared by coating a mixture of the nanosheet sample, carbon black and PVDF (85:10:5 wt%) in N‐methyl‐2‐pyrrolidone onto gold‐sputtered polyethylene terephthalate (PET) substrates.

**Figure 6:**
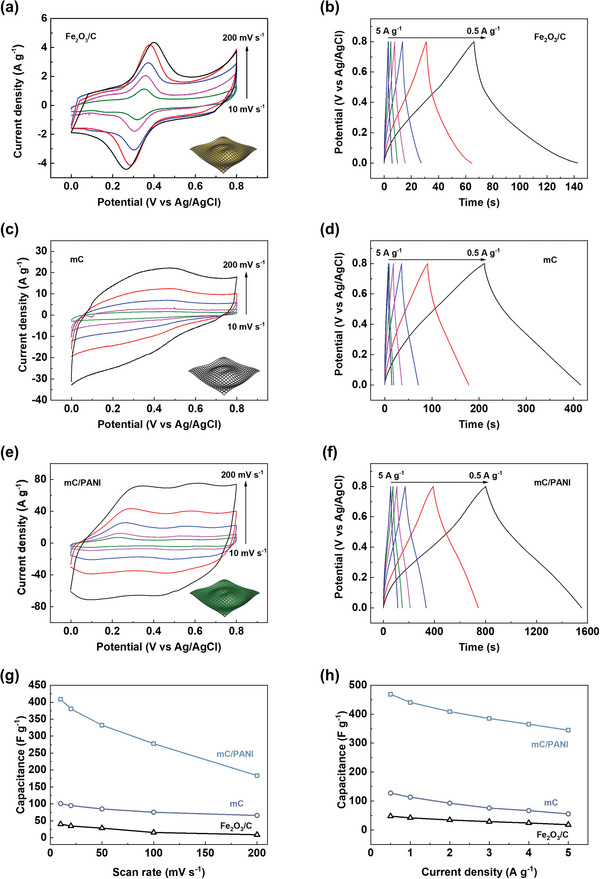
CV curves of a) Fe_2_O_3_/C, c) mC, and e) mC/PANI nanosheets obtained by varying the scan rate from 10 to 200 mV s^−1^. Insets illustrate the samples as depicted in Figure 1. GCD curves of b) Fe_2_O_3_/C, d) mC, and f) mC/PANI nanosheets measured by changing the current density from 0.5 to 5 A g^−1^. The electrochemical properties were studied using a three‐electrode system composed of active materials, Pt wire and Ag/AgCl in 1 m KOH or H_2_SO_4_ electrolyte solution. The base electrolyte was used for Fe_2_O_3_/C to avoid the degradation of the metal oxide nanocrystals and the acid solution was applied to the mC and mC/PANI nanosheets. Capacitance values calculated from g) the CV and h) GCD curves are plotted for comparison.

**Figure** **6** shows the cyclic voltammetry (CV) and galvanostatic charge–discharge (GCD) curves of the nanosheets. In Figure 6a, CV curves of Fe_2_O_3_/C exhibit a redox reaction ≈0.35 V, associated with the reversible Fe^2+^/Fe^3+^ transition (Fe_2_O_3_ + OH^−^ ⇆ Fe_2_O_3_OH + e^−^).^[^ ^67^ ^]^ Although iron oxides themselves are not conductive materials, the conductive carbon matrix enables the pseudocapacitive contribution and allows for the operation potential window ranging from 0 to 0.8V (vs Ag/AgCl), reaching the capacitance of 40.9 F g^−1^ at a scan rate of 10 mV s^−1^. However, the cathodic and anodic peaks move away from the center with increasing scan rate and the current becomes nearly saturated with 22.5% retention of capacitance (9.2 F g^−1^) at 200 mV s^−1^. The GCD curves confirm the rate capability of the Fe_2_O_3_/C sample in a comparable capacitance range (Figure 6b). Capacitance calculated from GCD curves drops from 47.6 to 18.2 F g^−1^ after increasing the current density from 0.5 to 5 A g^−1^, which is a 38.2% retention of capacitance. The capacitance generally decreases with increasing scan rate and current density, but the significant capacitance drop suggests that the capping carbon may not deliver charges quickly enough into the nanocrystal cores under fast kinetic conditions.

The mC nanosheets showed superior capacitive properties. The specific current in the CV curves increased after removal of the relatively heavy nanocrystal cores (Figure 6c). However, the curves did not exhibit rectangular EDLC shapes from pure carbon. Redox peaks appear with much smaller current signals than those of the Fe_2_O_3_/C sample. Because no crystal structure can be found from XRD, the redox reactions result from the small amount of residual Fe^3+^ ions remaining after the etching process. The insertion of redox‐active species, including hydroquinone,^[^ ^68^ ^]^ potassium iodide,^[^ ^69^ ^]^ and decamethylferrocene^[^ ^70^ ^]^ has been studied to improve capacitance, but the presence of residual salt in mC nanosheets provides an additional pseudocapacitive contribution at the electrode/electrolyte interface. Capacitance calculated from the CV curves reaches 101.0 F g^−1^ at 10 mV s^−1^ with a 55.5% retention at 200 mV s^−1^. The GCD curves show hybrid capacitive features with an improved rate capability (Figure 6d). The slope change in the GCD curves indicates a Faradaic reaction caused by the redox‐active Fe^3+^ salts. The capacitance values from GCD curves are from 127.5 to 55.7 F g^−1^ at the current densities of 0.5 to 5 A g^−1^, which is a 43.7% retention.

The capacitive properties were further improved by adding PANI to the mC nanosheets. The largest CV loop area and highest current response demonstrated the highest energy storage capability of the mC/PANI nanosheets (Figure 6e). Two redox peaks clearly appear in the CV curves, arising from the Faradaic transitions of leucoemeraldine/emeraldine and emeraldine/pernigraniline.^[^ ^39^, ^40^ ^]^ The combined pseudocapacitance and EDLC result in the high capacitance of 408.8 F g^−1^ at 10 mV s^−1^. The GCD curves of the mC/PANI sample also show the hybrid effect on the capacitance (Figure 6d). The three different slopes in the GCD curves are consistent with the two redox reactions and EDLC contribution. The capacitance calculated from the GCD curves attains 469.2 F g^−1^ at 0.5 A g^−1^ with 73% retention at 5 A g^−1^. For comparison, all the capacitance values calculated from the CV and GCD curves are plotted in Figure 6g,h. In particular, the high capacitance and rate capability of the mC/PANI nanosheets reflect their high conductivity and porous structure, which enables fast charge supply with a short electrolyte diffusion distance.

### 2.3 Electrochemical Performance of the Symmetric Supercapacitor

Symmetric supercapacitors were fabricated by sandwiching a PVA/H_2_SO_4_ gel electrolyte between electrodes. mC and mC/PANI nanosheets were chosen as the electrode materials because of their high energy‐storage capability in an acid electrolyte solution. When the two electrodes have the same capacitance, a maximized device capacitance can be achieved with a quarter of the half‐cell capacitance, considering the mass of each electrode (1/*C*=1/*C*_+_ + 1/*C*_−_). Thus, the same mass of active materials was loaded onto the positive and negative electrodes for the charge balance of supercapacitors. The electrochemical measurements were performed multiple times to confirm the charge balance with reproducibility and we presented the best‐performing data.

**Figure 7:**
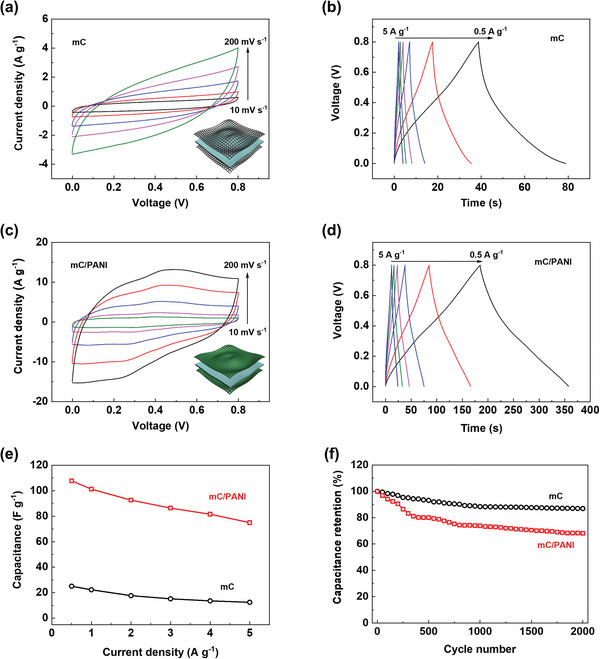
Electrochemical performance of symmetric supercapacitors fabricated by sandwiching a PVA/H_2_SO_4_ gel electrolyte with active materials. CV curves of a) mC//mC and c) mC/PANI//mC/PANI devices obtained by changing the scan rate from 10 to 200 mV s^−1^. Insets illustrate the architecture of the symmetric supercapacitors. GCD curves of b) mC//mC and d) mC/PANI//mC/PANI devices measured by varying the current density from 0.5 to 5 A g^−1^. e) Device capacitance calculated from the GCD curves. f) Cycling test of the supercapacitors obtained at a current density of 0.5 A g^−1^.

To evaluate the performance of the symmetric supercapacitors, their electrochemical features were studied using CV and GCD tests. In **Figure** **7**a, the CV curves of the mC//mC supercapacitor show that the charge storage mechanism mainly relies on the EDLC in the two‐electrode system. The Faradaic reaction of residual Fe ions, observed in the three‐electrode configuration, was hardly detected because of the relatively slow ion transport in the solid‐state gel electrolyte. The curves are also tilted by increasing the scan rate from 10 to 200 mV s^−1^, decreasing the capacitance from 21.9 to 8.2 F g^−1^ with 37.4% retention (Figure S6, Supporting Information). The disappearance of redox peaks and tilted shape indicate that the symmetric device made with mC exhibit relatively resistive behaviors. The GCD curves of the mC//mC device are shown in Figure 7b. Capacitance calculated from the GCD curves reaches 25.1 F g^−1^ at 0.5 A g^−1^_._ The device capacitance, which cannot exceed a quarter of half‐cell capacitance, reached 78.7% of the maximum theoretical value. With an increased current density of 5 A g^−1^, the device retains 49.8% of capacitance with 12.5 F g^−1^.

Improved energy storage was achieved using a symmetric supercapacitor composed of mC/PANI nanosheets. Owing to the increased conductivity, both EDLC and pseudocapacitive contributions are clearly visible in the CV curves in Figure 7c. The curve shapes are slightly deformed compared to those from the three‐electrode system because of slower electrolyte transport. However, two redox peaks clearly appeared, indicating that the Faradaic transformations of PANI occurred even with the solid‐state polymer gel electrolyte. Capacitance from the CV curves changes from 92.7 to 44.8 F g^−1^ by varying the scan rate from 10 to 200 mV s^−1^, which is 48.3% capacitance retention (Figure S6, Supporting Information). The GCD curves also showed prolonged charging and discharging times (Figure 7d). Capacitance calculated from the GCD data changes from 107.8 to 75.0 F g^−1^ upon increasing the current density from 0.5 to 5 A g^−1^ with 69.6% of retention. The device capacitance attains 91.9% of the maximum theoretical value at 0.5 A g^−1^. The capacitance difference between the mC and mC/PANI supercapacitors is shown in Figure 7e. The device capacitance values increased ≈4‐fold with the PANI coating on the mC nanosheets owing to the high conductivity and porous structure. However, mC and mC/PANI supercapacitors exhibited 86.9% and 68.1% of capacitance retention after 2000 cycles operated at a current density of 0.5 A g^−1^ (Figure 7f). Cyclability depends on the primary energy storage mechanisms. The mC device mainly relies on the stable ion adsorption and desorption process, whereas the mC/PANI device involves Faradaic reactions that could degrade the electrodes with repeated cycles. Although the latter cannot outperform the former mechanism in terms of cyclability, the redox damage is highly relaxed by the porous structure to deliver a capacitance of 73.4 F g^−1^ after 2000 cycles of the GCD test. Furthermore, Coulombic efficiency values of the symmetric supercapacitors fabricated with mC and mC/PANI nanosheets were calculated (Figure S7, Supporting Information). The efficiency of the mC/PANI device slightly decreased with decreasing the current density, involving more redox reactions that could degrade the electrode. Such results indicate that a stable EDLC mechanism provided by mC gives ≈100% efficiency, while a pseudocapacitive mechanism may degrade the electrode, but minimized by mesoporous nature of mC/PANI.

**Figure 8:**
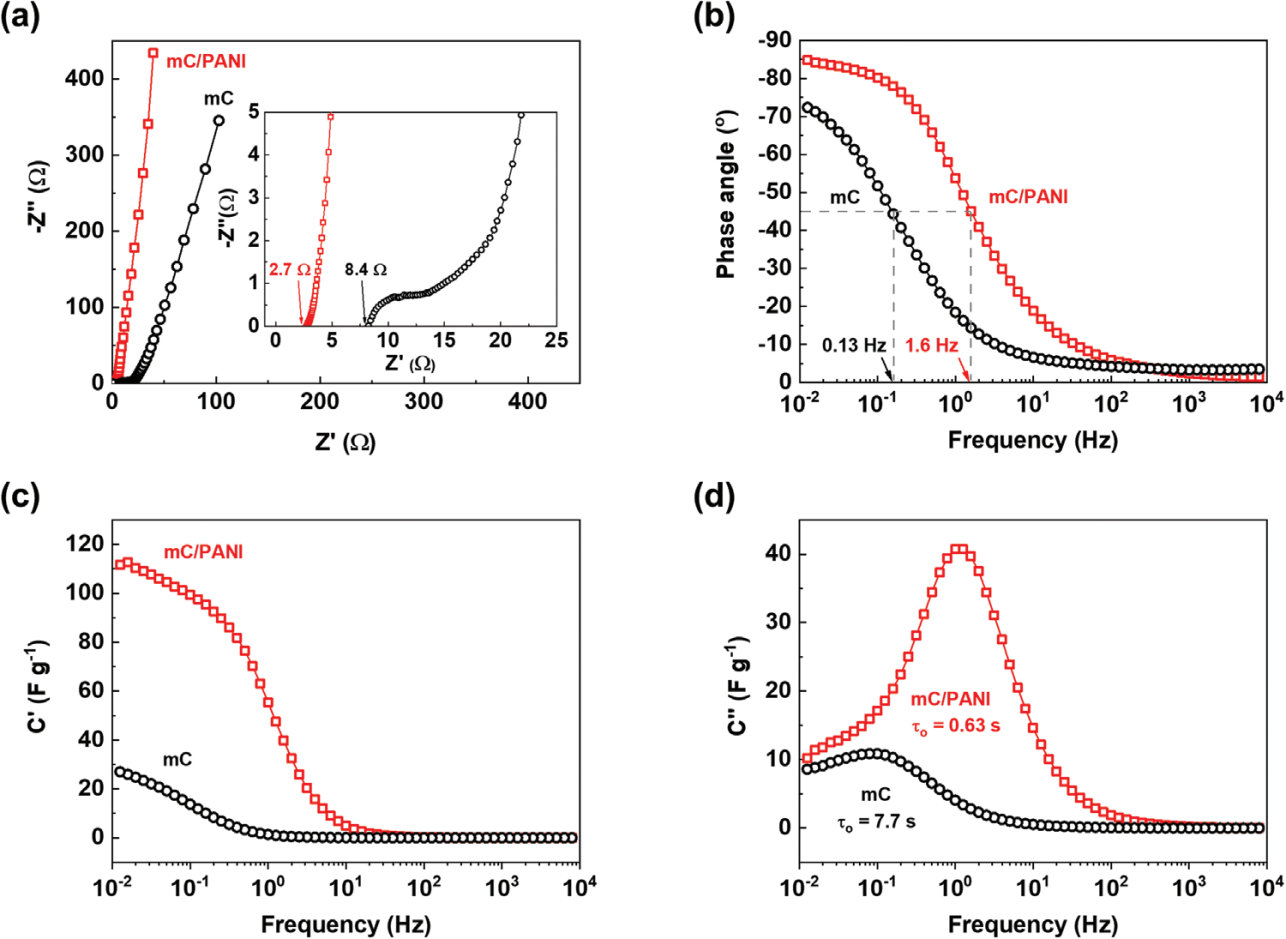
EIS analysis of the symmetric supercapacitors made with mC and mC/PANI nanosheets. Data were recorded by applying an alternating voltage amplitude of 10 mV in the frequency range from 0.012 to 8000 Hz. a) Nyquist plot of the supercapacitor devices shows a steep increase in the imaginary part of impedance relative to the real part of impedance. Inset is the magnified Nyquist plot showing the ESR of 8.4 and 2.7 Ω for the mC and mC/PANI devices. b) Bode phase angle of the devices at the scanned frequency. The frequency values that match the phase angle of −45° are 0.13 and 1.6 Hz for the mC and mC/PANI devices. c) Real part and d) imaginary part of capacitance of the devices at the alternating voltage frequency. The time constant (*τ*_0_) values of mC and mC/PANI devices are 7.7 and 0.63 s, respectively, which are consistent with the frequency corresponding to the phase angle of −45°.

The response speed of the devices was examined using electrochemical impedance spectroscopy (EIS). The impedance was tested in the frequency range of 0.012−8000 Hz with an alternating voltage amplitude of 10 mV. **Figure** **8**a shows Nyquist plots of the symmetric supercapacitors made with mC and mC/PANI nanosheets. In the high‐frequency regime, a resistive response was observed, as represented by the equivalent series resistance (ESR) and charge transfer resistance (*R*_ct_). The ESR is associated with the total resistance, including the intrinsic resistance of the substrates and the contact resistance at the interface between the active materials and the current collector.^[^ ^71^ ^]^ *R*_ct_ involves the Faradaic resistance at the interface between the electrode and electrolyte.^[^ ^72^ ^]^ The ESR values of mC and mC/PANI supercapacitors are 8.4 and 2.7 Ω, respectively. Owing to the high conductivity and porous channels for ion transport, *R*_ct_ is negligibly small for the mC/PANI device, while a semicircle is observed for the mC device with an *R*_ct_ of 21.6 Ω. In the low‐frequency regime, both devices exhibit capacitive behavior with a sharp increase in the imaginary part of impedance, *Z*′′(*w*), relative to the real part of impedance, *Z*′(*w*); a vertical increase with a phase angle of −90° represents ideal capacitor charging.^[^ ^73^ ^]^ The higher slope of the mC/PANI device indicates faster ion diffusion. The Warburg element can be used as a guide to describe the ion diffusion resistance and is apparent at a phase angle of −45°. The frequency corresponding to the −45° phase angle is the transition regime between resistive and capacitive behaviors.^[^ ^74^ ^]^ Figure 8b shows the Bode phase plot of the devices. The midrange frequency of mC and mC/PANI devices occurs at 0.13 and 1.6 Hz, respectively, which confirms the reduced diffusion resistance by the PANI coating on mC nanosheets.

Complex capacitance analysis further reveals the frequency‐dependent response of the devices (Figure 8c,d). The real part of the capacitance, *C*′, increases with decreasing frequency and then tends to level off as the frequency approaches zero. The low‐frequency value of *C*′ corresponds to the capacitance measured during the constant‐current discharge.^[^ ^75^ ^]^ The mC and mC/PANI devices exhibit *C*′values value of 27.0 and 111.6 F g^−1^ at a low frequency of 0.012 Hz, which is comparable to the result obtained at ≈1 A g^−1^ in Figure 7g. The imaginary part of the capacitance, *C*′′, corresponds to energy dissipation by an irreversible process.^[^ ^74^ ^]^ The maximum value of *C*′′ occurs at frequency *f*_0_, defining a time constant *τ*_0_ = 1/*f*_0_. Half of the low‐frequency *C*′ is achieved at *τ*_0_ with a phase angle of −45°. Thus, the time constant represents a transition between resistive behavior for frequencies higher than 1/*τ*_0_ and capacitive behavior for frequencies lower than 1/*τ*_0_.^[^ ^76^ ^]^ The time constant (*τ*_0_) values for the mC and mC/PANI devices are 7.7 and 0.63 s, respectively, which is consistent with the result obtained from the Bode phase plot in Figure 8b. The shorter time constant of the mC/PANI supercapacitor indicates a faster response to capacitive energy storage. The fast charging and discharging properties of the mC/PANI device resulted from the improved conductivity and porous structure that facilitated charge and electrolyte transport.

**Figure 9:**
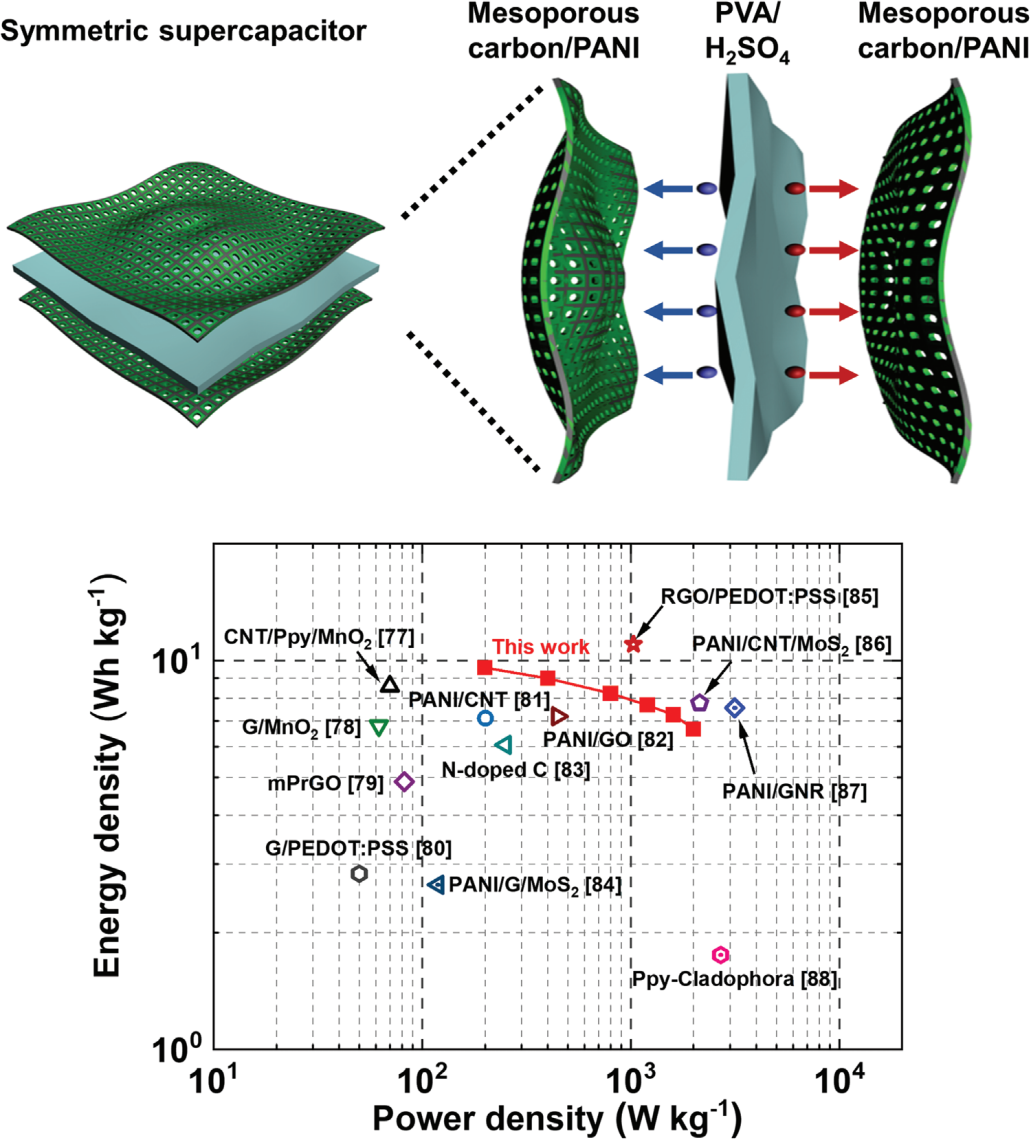
Ragone plot of the symmetric supercapacitor device made with mC/PANI nanosheet. The energy density and power density of the device are compared with various symmetric supercapacitors. The results are higher than the values obtained from many other symmetric supercapacitors and are attributed to the improved conductivity and porous structure. The image on the top illustrates the symmetric supercapacitor architecture made with mC/PANI and PVA/H_2_SO_4_ gel electrolyte.

**Figure** **9** shows the Ragone plot of the symmetric supercapacitor fabricated with mC/PANI nanosheets. The energy and power densities of the device are compared to those of other symmetric supercapacitors.^[^ ^77^, ^78^, ^79^, ^80^, ^81^, ^82^, ^83^, ^84^, ^85^, ^86^, ^87^, ^88^ ^]^ Energy density refers to the amount of energy that can be stored, whereas power density refers to how quickly energy can be discharged. Thus, efficient energy storage devices should provide a high energy density without a significant reduction in the power density, and vice versa. The mC/PANI device exhibits gravimetric energy density and power density of 9.59 Wh kg^−1^ and 200.1 W kg^−1^, respectively, at a current density of 0.5 A g^−1^. As the current density increases to 5 A g^−1^, the energy density decreases to 6.67 Wh kg^−1^ and power density increases to 2000 W kg^−1^. Thus, the device delivers a sufficiently high energy density, even at a high power density. Notably, the performance was achieved by the relatively small amount of PANI content of 37.1 wt% to many other PANI‐based symmetric supercapacitors. (The PANI contents of PANI/GNR, PANI/CNT, and PANI/GO in Figure 9 are 97.5, 94, and 81 wt%, respectively.) Also, these values are higher than those obtained from many other symmetric supercapacitors and are attributed to the improved conductivity and porous structure of mC/PANI, which enables fast charge and electrolyte transport. Therefore, our synthetic route for conducting polymer deposition on mesoporous carbon surfaces is a promising approach for high‐performance supercapacitors.

## 3 Conclusions

PANI‐capped mC nanosheets were successfully synthesized with high conductivity and porosity by VDP. The carbon template was prepared by etching the Fe_2_O_3_ nanocrystals embedded in a carbon matrix obtained by heating iron oleate in a NaCl matrix. The optimized heating rate was found to produce an ordered assembly of cubic Fe_2_O_3_ nanocrystals with an average edge length of 40 nm. Complete removal of the nanocrystals was achieved using aqua regia, leaving the ordered mesoporous carbon frameworks. Evaporated aniline monomers were then slowly polymerized on the mC nanosheets pretreated with FeCl_3_ as an initiator. PANI partially filled the carbon pores and maintained the porous structure. The incorporation of conducting polymers also improved the conductivity of mesoporous carbon, wherein many defect sites were generated during the etching step. The improved conductivity with the retention of pores led to efficient hybrid energy storage mechanisms of EDLC and pseudocapacitance. In a half‐cell configuration, the mC/PANI nanosheets exhibited a high capacitance of 469.2 F g^−1^ at a current density of 0.5 A g^−1^, which is ≈3.7 times higher than that when using only mC nanosheets. When the mC/PANI nanosheets were assembled as a symmetric supercapacitor, the device capacitance reached 107.8 F g^−1^ and the cycle test demonstrated 73.8% capacitance retention after 1000 cycles at 0.5 A g^−1^. The high capacitance and cyclability was attributed to the fast ion and charge transport from the conductive and porous material surface, as demonstrated by the time constant (*τ*_0_) values of 0.63 s from the EIS analysis. The rapid transition from resistive to capacitive regimes allows for efficient energy storage. The corresponding energy density and power density were 9.59 Wh kg^−1^ and 200.1 W kg^−1^, respectively, at a current density of 0.5 A g^−1^, which are higher than the values obtained for majority of the reported symmetric supercapacitors.

## 4 Experimental Section

### Chemicals

Iron(III) chloride hexahydrate (FeCl_3_·6H_2_O, 97%), iron(III) chloride (FeCl_3_, 97%), aniline (>99.5%), sodium chloride, N‐methyl‐2‐pyrrolidone (anhydrous, 99.5%), nitric acid (70%), hydrochloric acid (37.5%), and hexane (anhydrous, 95%) were purchased from Sigma‐Aldrich. Absolute ethanol was obtained from Fisher Scientific. Sodium oleate (95%) was purchased from Tokyo Chemical Industry (TCI). All the chemicals were used without further purification.

### Synthesis of Iron Oxide/Carbon (Fe_2_O_3_/C) Nanosheets

FeCl_3_·6H_2_O (0.36 g, 1.33 mmol) and sodium oleate (1.22 g, 4 mmol) were mixed in a solution of ethanol (3 mL), water (2 mL), and hexane (5 mL) under vigorous stirring overnight. The mixture was heated to 65 °C and maintained at this temperature for 4 h. The upper layer, containing the iron‐oleate complex and hexane, was washed three times with distilled water (5 mL) using a separatory funnel and the solution was evaporated using a rotary evaporator. The waxy solid of the iron‐oleate complex (0.3 g) was mixed with NaCl powder (10 g) using a mortar and pestle. The mixture was heated to 700 °C at a heating rate of 10 °C min^−1^ under nitrogen flow and the temperature was maintained for 3 h. After cooling to room temperature, the product was washed with water to dissolve NaCl salt and dried in a vacuum oven at 100 °C.

### Synthesis of mC Nanosheets

The iron oxide/carbon nanosheets were immersed in an acidic solution of HCl (3 mL) and nitric acid (1 mL) for 1 week to completely remove the iron oxide. The product was isolated by centrifugation at 8000 rpm for 5 min. The supernatant was discarded and the mesoporous carbon product was rinsed thoroughly with distilled water.

### Synthesis of Mesoporous Carbon Coated with Polyaniline (mC/PANI) Nanosheets

PANI was deposited onto mC nanosheets using VDP. mC nanosheets (0.2 g) were stirred in an FeCl_3_ aqueous solution (0.1 m, 15 mL) for 2 h. The FeCl_3_‐soaked nanosheets were collected by centrifugation at 8000 rpm for 10 min and residual FeCl_3_ was removed by absorption with disposable sorbents three times. For complete drying, the Fe‐ion‐adsorbed nanosheets were dried overnight in a vacuum oven. The product was placed in a reactor equipped with a sealing apparatus and a monomer reservoir. The reactor was evacuated at room temperature using a Schlenk line. Aniline (0.1 mL) was carefully injected into the monomer loading reservoir and the reactor was placed in an oven at 60 °C for 6 h. The final product was collected and stored in a vacuum oven.

### Materials Characterization

Field‐emission scanning electron microscopy images were acquired with a JSM‐6701F microscope at an accelerating voltage of 10 kV using an in‐lens detector. SEM samples were deposited on Si substrates using copper tape to prevent charging. TEM images were obtained using a JEM‐2100 instrument at an accelerating voltage of 80 kV. HR‐TEM was performed using a JEM 2010 instrument operating at 200 kV. The TEM samples were prepared by drop‐casting nanosheets from dilute dispersions in ethanol onto continuous carbon‐coated copper mesh grids (Electron Microscopy Science). The TEM grids were placed in a vacuum oven at 70 °C to reduce contamination.

XRD was performed using a Rigaku Smart Lab X‐ray diffractometer with Cu K*α* radiation (*λ* = 1.541 Å), operated at 40 kV and 40 mA. Data were collected from the nanosheets on glass substrates at an angle rotation rate of 10° min^−1^. XPS was performed using a Sigma Probe (Thermo‐VG, UK) spectrometer with monochromatic Al K*α* radiation (*hν* = 1486.5 eV). Samples were prepared by drop‐casting nanosheets in ethanol dispersion onto a conductive Boron‐doped silicon wafer (GlobiTech, 0.01–0.02 Ω cm). Deconvolution was performed by fitting the peaks to a Gaussian distribution function. Sample charging was corrected by shifting the C 1s peaks to a value expected for hydrocarbons at 284.8 eV.

Attenuated total reflectance Fourier‐transform infrared spectra were obtained using a Thermo Scientific Nicolet 6700 spectrometer. The samples were drop‐cast onto crystal plates of the ARK module. Spectra were acquired using 512 scans at a resolution of 4 cm^−1^. Raman spectra were recorded using a Horiba Jobin Yvon LabRam Aramis spectrometer with 532 nm laser excitation. Samples were deposited on 2.5 × 2.5 cm^2^ Al foil substrates and spectra were acquired for 10 s. N_2_‐sorption isotherms were obtained using a Micromeritics ASAP 2010 (accelerated surface area and porosimetry system) to obtain surface area using the BET method. TGA was performed using a TGA 2050 analyzer (TA Instruments). Samples on crucibles were heated to 700 °C at a heating rate of 10 °C min^−1^.

### Electrochemical Measurements

Electrochemical analysis was carried out using a three‐electrode system consisting of the nanosheet‐based working electrode, an Ag/AgCl electrode as the reference electrode and a Pt wire as the counter electrode. The working electrodes were prepared by coating a mixed paste of nanosheets, carbon black and PVDF (85:10:5 wt%) in N‐methyl‐2‐pyrrolidone on gold‐sputtered PET substrates. The three electrodes were immersed in 1 m KOH or H_2_SO_4_ electrolyte for electrochemical characterization. Symmetric supercapacitors were also fabricated to study actual device performance. The separator was prepared using a PVA/H_2_SO_4_ gel electrolyte. PVA (12 g) was heated in distilled water at 70 °C overnight, followed by degassing in a vacuum oven. The PVA solution was cast on glass slides and the dried film was peeled off using a spatula. The thin PVA film was immersed in a 1 m H_2_SO_4_ aqueous solution and sandwiched between the two electrodes.

CV and GCD measurements were conducted on an electrochemical workstation (Zahner Elektrik IM6). CV and GCD measurements were performed by varying the scan rate from 10 to 200 mV s^−1^ and the current density from 0.5 to 5 A g^−1^, respectively. Specific capacitance was calculated using the following equations^[^ ^67^ ^]^

(1) $$C\;=\;\frac{1}{m\;\times \;\nu \;\times \;\Delta V}\;\int_{V_{0}}^{V}I\;dV\;\left(\mathrm{from}\;\mathrm{CV}\;\mathrm{curves}\right)$$

(2) $$C\;=\;\frac{1}{m\;\times \;\Delta V}\;\int_{t_{0}}^{t}I\;dt\;\left(\mathrm{from}\;\mathrm{GCD}\;\mathrm{curves}\right)$$

where *C* is the specific capacitance, *I* is the discharging current, *ν* is the scan rate, *V* is the voltage, Δ*V* is the operating voltage window, and *m* is the total mass, including the additives. The Coulombic efficiency (*η*) is defined by the output charge relative to the input charge, as follows ^[^ ^68^ ^]^

(3) $$\eta \;=\frac{q_{d}}{q_{c}}\;\times 100\;\left(\%\right)$$

where *q*_c_ and *q*_d_ are the total amount of charge and discharge, respectively. The specific energy density (Wh g^−1^) and power density (W g^−1^) generated from the GCD curves were calculated as follows^[^ ^67^ ^]^

(4) $$E\;=\;\frac{1}{2}C\Delta V^{2}$$

(5) $$P\;=\;\frac{E}{\Delta t}$$

where *E* is the energy density and *P* is the power density, respectively.

EIS measurements were performed using an electrochemical workstation (Zahner Elektrik IM6). Impedance values were recorded by applying an alternating voltage of 10 mV in the frequency range of 0.012–8000 Hz. The resulting impedance values are associated with the resistance (*R* (*w*)) and capacitance (*C* (*w*)) terms, which are functions of the alternating voltage frequency *w*. *C* (*w*) is defined by the complex expression

(6) $$C\;\left(w\right)=C^{\prime }\;\left(w\right)-iC^{\prime \prime }\left(w\right)$$

The real and imaginary parts of the complex capacitance can be obtained using frequency‐dependent impedance values as follows^[^ ^70^ ^]^

(7) $$C^{\prime }\;\left(w\right)=\;\frac{-Z^{\prime \prime }\left(w\right)}{w{\left|Z\left(w\right)\right|}^{2}},\;C^{\prime \prime }\;\left(w\right)=\;\frac{Z^{\prime }\left(w\right)}{w{\left|Z\left(w\right)\right|}^{2}}$$

where *Z*′(*w*) and *Z*′′(*w*) are the real and imaginary parts of the impedance (*Z*(*w*)) and *C*′(*w*) and *C*′′(*w*) are the real and imaginary parts of the impedance (*C* (*w*)), respectively.

### Statistical Analysis

The illustration models were constructed on 3D Max software (Autodesk, San Rafael, CA, USA). The TEM and HR‐TEM images were analyzed on Digital Micrography software (Gatan Inc, Pleasanton, CA, USA). The SEM images were processed using ImageJ software. The size of nanocrystals was evaluated by averaging 100 particles and presented as mean ± SD. The XRD, XPS, Raman, and FTIR were analyzed on Jade software (Materials Data, Livermore, CA, USA), CasaXPS software (Casa software Ltd.), Labspec software (Horiba, Kyoto, Japan), and OMNIC software (Thermo Fisher Scientific Inc, Waltham, MA, USA), respectively. CV, GCD, and EIS were analyzed on an electrochemical workstation (Zahner Elektrik IM6). The reproducibility was confirmed by measuring the electrochemical data multiple times to obtain a charge‐balanced device and the best‐performing data were included. The gravimetric values were estimated by weighing the total mass including active material, binder, and carbon black. Results were analyzed on OriginPro software (Origin Lab, Northampton, MA, USA).

## Conflict of Interest

The authors declare no conflict of interest.

## Supporting information

Supporting Information

## Data Availability

Research data are not shared.
