# Supplementary material for: Polyaniline‐Coated Mesoporous Carbon Nanosheets with Fast Capacitive Energy Storage in Symmetric Supercapacitors
Source: Adv Sci (Weinh). 2023 May 10;10(21):2301923. doi: 10.1002/advs.202301923 (PMC10375140; doi:10.1002/advs.202301923)
Supplement: Supplementary file 1 — Supporting Information [file ADVS-10-2301923-s001.pdf]

ADVANCED SCIENCE
Open Access

Supporting Information

for *Adv. Sci.*, DOI 10.1002/advs.202301923

Polyaniline-Coated Mesoporous Carbon Nanosheets with Fast Capacitive Energy Storage in Symmetric Supercapacitors

*Jungchul Noh, Suk Jekal and Chang-Min Yoon**

Supporting Information for

Polyaniline-Coated Mesoporous Carbon Nanosheets with Fast Capacitive Energy Storage in Symmetric Supercapacitors

Jungchul Noh[†], Suk Jekal[‡], and Chang-Min Yoon[‡*]

[†]McKetta Department of Chemical Engineering, The University of Texas at Austin, Austin, TX, 78712, USA.

[‡]Department of Chemical and Biological Engineering, Hanbat National University, Daejeon, 34158, Republic of Korea

[*]Corresponding author: (T) +82-42-821-1528; cmyoon4321@hanbat.ac.kr

List of Figures

- **Figure S1.** TEM image of Fe_2O_3/C synthesized by the thermal decomposition of iron oleate complex without NaCl addition.
- **Figure S2.** TEM images of Fe_2O_3/C synthesized by varying the heating rate.
- **Figure S3.** N_2-sorption isotherms of Fe_2O_3/C, mC and mC/PANI nanosheets.
- **Figure S4.** HR-TEM image of Fe_2O_3/C showing the lattice spacing.
- **Figure S5.** Selected area electron diffraction patterns of Fe_2O_3/C, mC and mC/PANI nanosheets.
- **Figure S6**. Capacitance values of the mC and mC/PANI supercapacitors with varying the scan rate
- **Figure S7.** Coulombic efficiency of the symmetric supercapacitors made with mC and mC/PANI nanosheets.

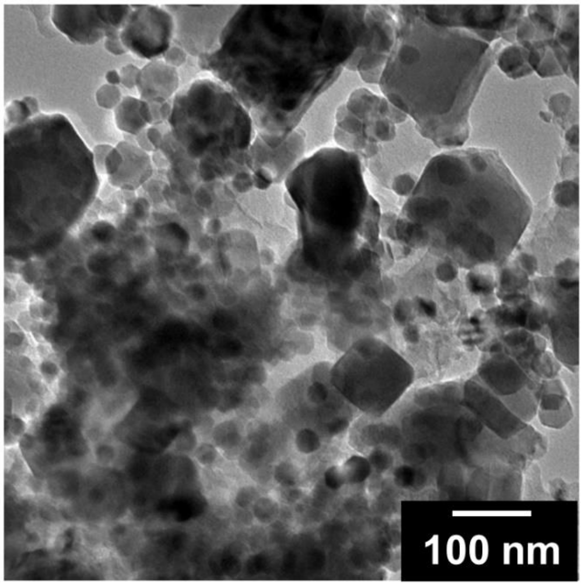

Figure S1. TEM image of iron oxide nanocrystals embedded in carbon synthesized by heating an iron-oleate complex (0.3 g) to 700 °C for 3 h at a heating rate of 10 °C min^{-1} under nitrogen atmosphere. The thermal decomposition of iron-oleate complex without NaCl produces iron oxide nanocrystals with a highly broad size distribution of 10–200 nm and irregular shape.

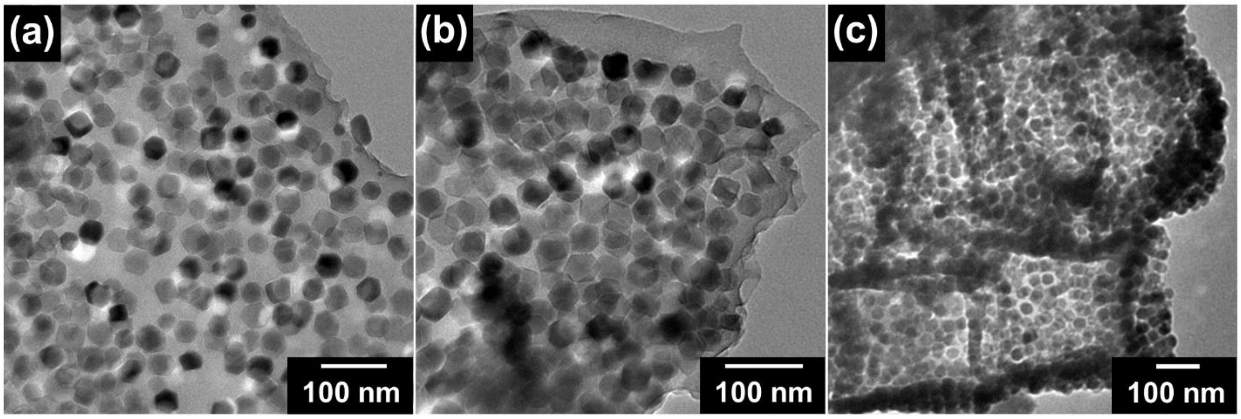

Figure S2. TEM images of iron oxide nanocrystals embedded in carbon synthesized by heating an iron-oleate complex (0.3 g) in NaCl powder matrix (10 g) to 700 °C for 3 h under nitrogen atmosphere at heating rates of (a) 2 °C min^{-1}, (b) 5 °C min^{-1} and (c) 20 °C min^{-1}.

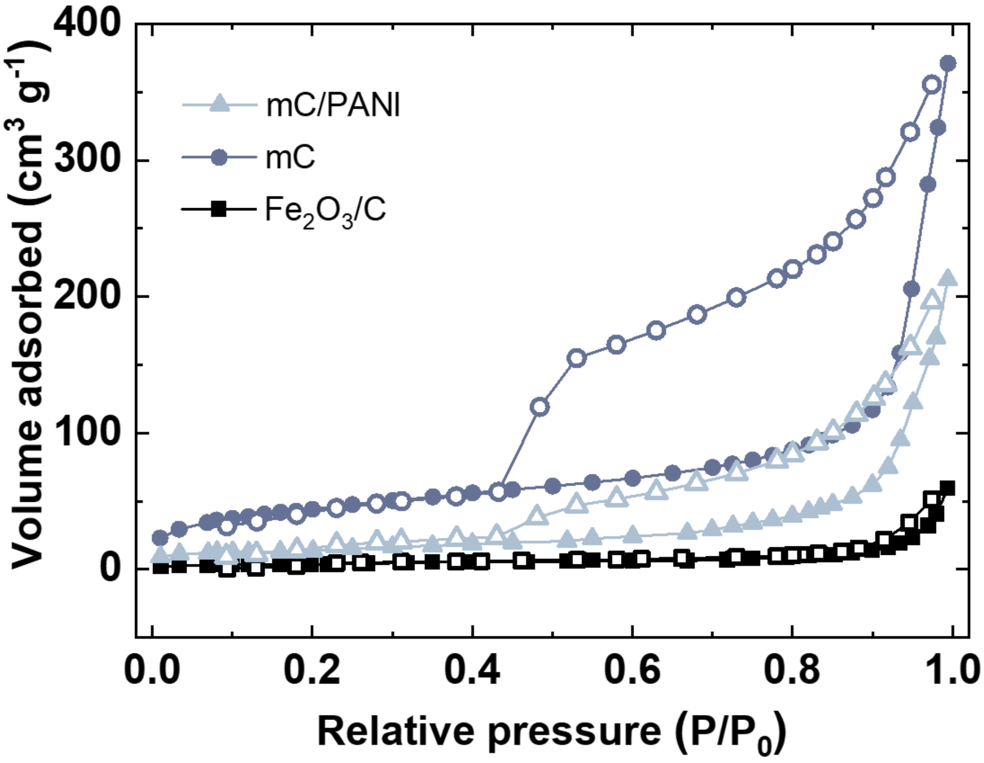

Figure S3. N_2-sorption isotherms of Fe_2O_3/C, mC and mC/PANI nanosheets obtained by using liquid nitrogen at –196 °C. Using BET analysis, the obtained surface area values of the Fe_2O_3/C, mC and mC/PANI nanosheets are 10.9, 161 and 46.0 $m^2\ g^{-1}$, respectively, indicating that PANI coating partially fills the mesopores of carbon.

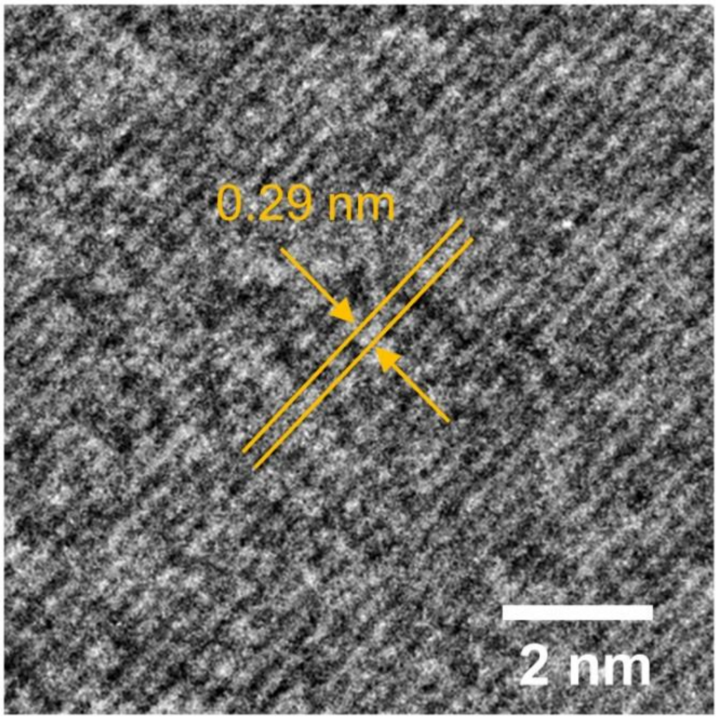

Figure S4. HR-TEM image of the Fe_2O_3/C sample with high magnification. The lattice spacing of 0.29 nm corresponds to the distance between (220) planes of maghemite γ-Fe_2O_3.

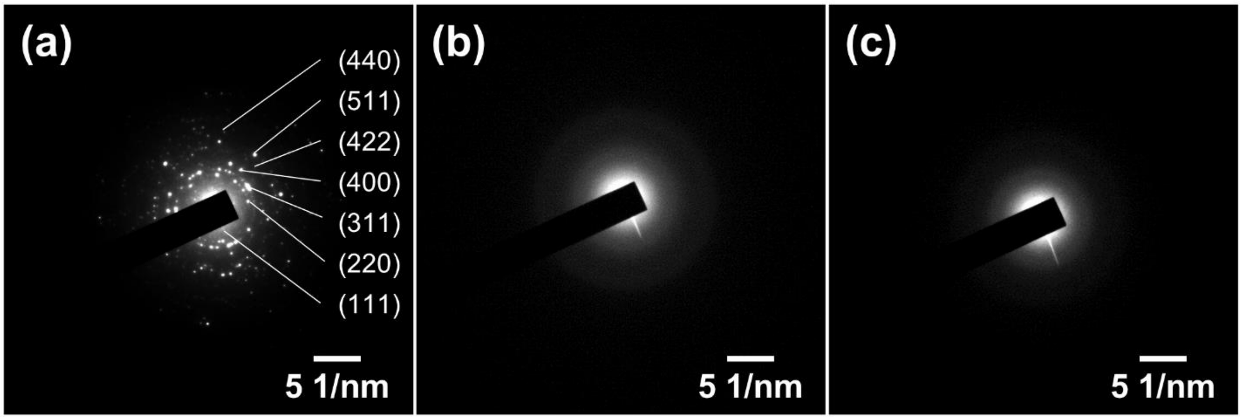

Figure S5. SAED patterns of (a) Fe_2O_3/C, (b) mC and (c) mC/PANI nanosheets. The diffraction pattern of iron oxide nanocrystals matches the *d*-spacings of maghemite γ-Fe_2O_3. The average (111), (220) and (311) d-spacings are 0.485, 0.297 and 0.253 nm, respectively, corresponding to a lattice constant of 0.839 nm, which is close to the literature value of 0.834 nm for magnetite γ-

Fe_2O_3. The spots in the SAED image reveal that the crystal facets of cubic Fe_2O_3 nanocrystals are randomly oriented. After the acid treatment and PANI coating, the spots are no longer observed, indicating the complete removal of nanocrystal cores.

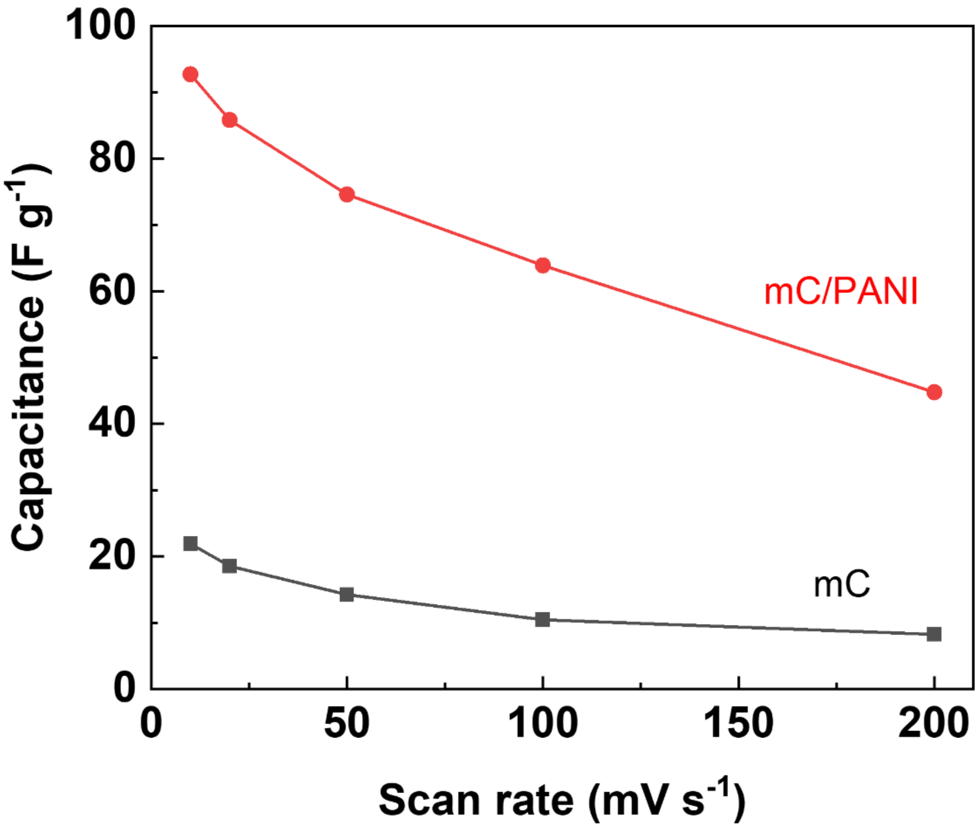

Figure S6. Capacitance values of the mC and mC/PANI symmetric supercapacitors with varying the scan rate from 10 to 200 mV s^{-1}. Capacitance of the mC device drops from 21.9 to 8.2 F g^{-1} with 37.4% retention. Capacitance of the mC/PANI device decreases from 92.7 to 44.8 F g^{-1} with 48.3% retention.

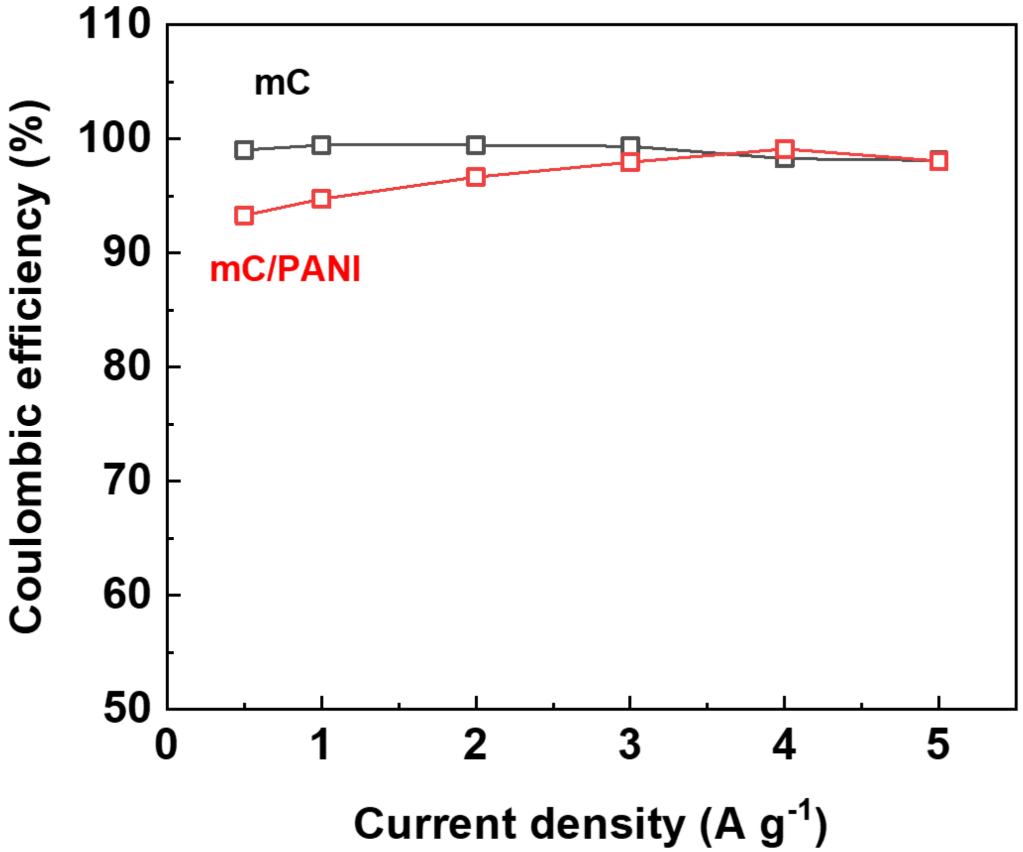

Figure S7. Coulombic efficiency of the symmetric supercapacitors made with mC and mC/PANI nanosheets, obtained by the ratio of charging and discharging time from the GCD curves in Figure 7. The mC device exhibits nearly 100% efficiency in the current density from 0.5 to 5 A g^{-1}. The efficiency of the mC/PANI device tends to decrease with decreasing the current density, involving more redox reactions that could degrade the electrode. The result indicates that a stable EDLC mechanism gives nearly 100% efficiency, while a pseudocapacitive mechanism that could degrade the electrode decreases the efficiency.
